# A Blow on the Ear with Peculiar Reflex Symptoms

**Published:** 1888-11

**Authors:** Alvin A. Hubbell

**Affiliations:** Professor of Diseases of the Eye, Ear, and Throat, in the Medical Department of Niagara University; Eye and Ear Surgeon to the Buffalo Hospital of the Sisters of Charity, etc.; 212 Franklin Street


### A BLOW ON THE EAR WITH PECULIAR REFLEX SYMPTOMS.

By ALVIN A. HUBBELL, M. D.,

Professor of Diseases of the Eye, Ear, and Throat, in the Medical Department of Niagara University; Eye and Ear Surgeon to the Buffalo Hospital of the Sisters of Charity, etc.

Various reflex phenomena, due to disease of the ear, or to the presence of foreign bodies in the external auditory canal, are well known. But those observed in the following case are new, at least so far as I can ascertain, and seem to me of sufficient interest to be placed on record:

R. P., aged fifteen, consulted me September 23, 1888. On the previous day he and a playmate quarreled, and the latter struck him on the left ear with his hand. The blow caused some pain and considerable impairment of hearing. Very soon afterwards it was noticed that there were peculiar convulsive movements of the lower jaw, by which it was quickly moved forwards, the mouth opening slightly at the same time. When I first saw him, these movements were very frequent, only a few seconds intervening between each one. They resembled those of chorea, but were limited to the lower jaw. The patient said he was unconscious of their taking place, and could not control them by will.

On examining the injured ear, I found that the drum-membrane was ruptured, was considerably red and tender, and a little blood was in the external auditory canal. The ticking of my watch, which should be heard five feet, was heard six inches.

No other symptoms were noticed. The lad was not robust, but appeared to be in good general health.

I cleansed the ear by syringing it gently with warm water, carefully dried it with absorbent cotton, inflated it with the air-bag, air and mucus escaping through the ruptured membrane, and applied to the latter powdered boracic acid.

The convulsive movements at once became less frequent and not as pronounced, and before leaving my office the interval between them was increased to several minutes.

The ear was treated for several days, by cleansing with warm water and applying boracic acid powder, when the perforation was healed. The convulsive movements gradually diminished in frequency until the fifth or sixth day, after which they entirely ceased. The patient was discharged October 11th, with hearing for the watch increased to three feet, and with a prospect of its being fully recovered.

As in chorea, these movements did not take place when the patient was asleep.

This was, without doubt, a case of reflex spasm, affecting especially the pterygoid muscles. The afferent channels of the reflex action were, evidently, the sensory branches of the trigeminus nerve distributed to the membrana tympani and tympanum, and the efferent channels were the motor fibres of the trigeminus going to the pterygoid muscles.

This case seems to me to be unique, and of peculiar physiological interest.

212 Franklin Street.

